# Ga-68 DOTATATE PET imaging in the management of head and neck paragangliomas: a potential game changer

**DOI:** 10.3389/fonc.2025.1675974

**Published:** 2025-11-18

**Authors:** Alaa Alduraibi, Noora Bin Essa, Ahmed A. Alruwaili, Jamshed Bomanji

**Affiliations:** 1 Department of Radiology, College of Medicine, Qassim University, Buraidah, Saudi Arabia; 2 Nuclear Medicine Department, Kuwait Cancer Control Center, Ministry of Health Kuwait, Kuwait, Kuwait; 3 Department of Radiology, King Faisal Specialist Hospital & Research Center, Riyadh, Saudi Arabia; 4 Institute of Nuclear Medicine, University College London, London, United Kingdom

**Keywords:** Diagnostic Sensitivity, Gallium-68 DOTATATE PET/CT, Head and Neck Paragangliomas, Metastatic Disease Detection, Neuroendocrine Tumors

## Abstract

**Background:**

Head and neck paragangliomas (HNPGs) are rare neuroendocrine tumors originating from neural crest cells, with some exhibiting malignant potential. Traditional imaging modalities, such as magnetic resonance imaging (MRI), often have limitations in detecting multifocal or metastatic disease, driving interest in more sensitive diagnostic approaches like Gallium-68 DOTATATE positron emission tomography/computed tomography (Ga-68 DOTATATE PET/CT).

**Methods:**

This retrospective study aimed to assess the effectiveness of Ga-68 DOTATATE PET/CT in the detection and management of HNPGs. Imaging data from 2015 to 2024 were reviewed, identifying four confirmed cases of HNPG. All patients underwent head and neck MRI and Ga-68 DOTATATE PET/CT scans. Data on imaging findings, treatment history, and clinical outcomes were collected and analyzed to compare the performance of Ga-68 DOTATATE PET/CT with other imaging techniques, including Iodine-123 meta-iodobenzylguanidine (I-123 MIBG) scintigraphy and Fluorine-18 fluorodeoxyglucose (F-18 FDG) PET/CT.

**Results:**

Ga-68 DOTATATE PET/CT demonstrated enhanced sensitivity in detecting both primary and metastatic lesions compared to MRI and other imaging modalities. It identified additional lesions not seen on MRI, confirmed local recurrence, and detected unsuspected metastatic sites, influencing clinical management. While Ga-68 DOTATATE PET/CT provided more diagnostic clarity than I-123 MIBG in one case, it also revealed additional metastatic sites in a patient with a succinate dehydrogenase (SDH) gene mutation, which were not detected by F-18 FDG PET/CT.

**Conclusion:**

Ga-68 DOTATATE PET/CT is a valuable imaging tool for evaluating HNPGs, with superior sensitivity in detecting both primary and metastatic lesions compared to conventional imaging. Its diagnostic advantages can significantly impact treatment planning and patient management. Integrating Ga-68 DOTATATE PET/CT into clinical guidelines for HNPG evaluation may enhance diagnostic accuracy. Further research with larger cohorts is warranted to confirm these findings and establish standardized interpretation criteria.

## Introduction

Paraganglioma (PG) is a neuroendocrine tumor that arises from neural-crest ganglia, originating from sympathetic and parasympathetic chains (1, 2). PGs can occur anywhere from the skull base to the pelvic floor as a result of the migration of embryonic cells, with an estimated 65–70% found in the head and neck region (2).

Although most head and neck paragangliomas (HNPGs) are diagnosed incidentally, they can present later in life with compressive symptoms affecting adjacent neurovascular structures, including bruit and cranial nerve deficits (3).

The vast majority of HNPGs are benign; however, rare malignant variants can occur, lacking definitive histopathological features even when using immunohistochemistry testing (2, 4). The only confirmed indicator of malignancy remains metastasis to non-neuroendocrine organs (1, 2, 4, 5). Head and neck magnetic resonance imaging (MRI) enhanced with gadolinium demonstrates a classical “salt and pepper” appearance in primary HNPG tumors (2) but does not offer whole-body evaluation for detecting multiple or metastatic lesions. Furthermore, the World Health Organization in its most recent updated guidelines advises that HNPGs, even after curative treatment, require routine follow-up because of their malignant potential, necessitating whole-body surveys (4). This, coupled with the lack of biochemical markers in nonfunctional HNPGs, makes diagnosis heavily reliant on imaging. A significant body of evidence in the literature demonstrates the superior performance of gallium-68 somatostatin receptor (SSRT) imaging using gallium-68 DOTATATE (Ga-68 DOTATATE) for various HNPG indications.

The purpose of this retrospective case series was to assess the role of Ga-68 DOTATATE in patients with HNPGs and compare its clinical impact with cross-sectional and other molecular imaging modalities.

## Methods

We conducted a retrospective review of our imaging database from 2015 to 2024 to identify cases of HNPG. We included all cases with confirmed HNPG, based on clinical, laboratory and imaging data. Patient selection criteria included documented diagnosis of HNPG, availability of imaging data, and complete treatment and outcome records.

### Imaging protocols

- MRI: All patients underwent head and neck contrast-enhanced MRI. The MRI protocol included T1-weighted, T2-weighted and post-gadolinium-enhanced T1-weighted sequences.
- PET Imaging: Ga-68 DOTATATE positron emission tomography (PET) imaging was performed for all patients. Case 1 was assessed with PET/MRI, while the remaining cases underwent PET/computed tomography (CT). The PET protocol involved scanning from the vertex to mid-thigh following the administration of 118–250 MBq; 3.2–6.75 mCi (mean 188.5 MBq; 5.1 mCi) of Ga-68 DOTATATE at 45–76 minutes (mean 62.75 minutes).

### Data collection

Data were collected from clinical records, MDT (Multi-disciplinary team meetings) notes and imaging results. The review included analysis of treatment history and clinical outcomes.

### Literature review

A comprehensive literature review was conducted using PubMed with the search term “paraganglioma” to contextualize our findings within the existing research.

## Results

We identified four cases of HNPG from our institutional imaging database, covering the period from 2015 to 2024. The patients were all male, aged between 59 and 70 years (median 64.5 years).

### Case 1

**Figure 1:**
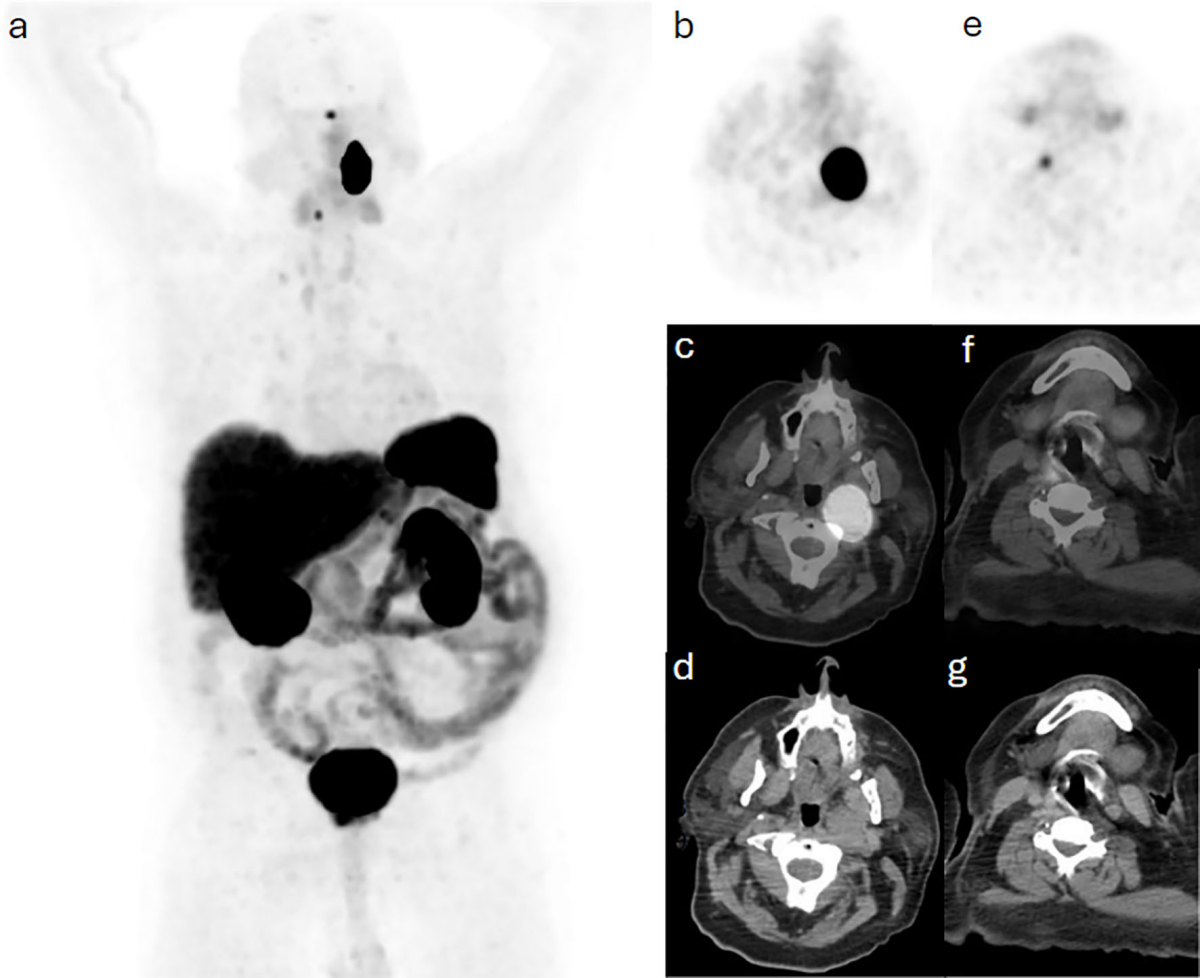
Case 1. **(a)** maximum intensity projection (MIP) demonstrating intense Ga68-DOTA-TATE uptake in the head and neck region bilaterally, which are further confined to a soft tissue lesion in the left retropharyngeal space and a smaller right focal tissue on trans-axial slices including **(b, e)** PET, **(c, f)** hybrid PET/CT and **(d, g)** CT images.

**Figure 2:**
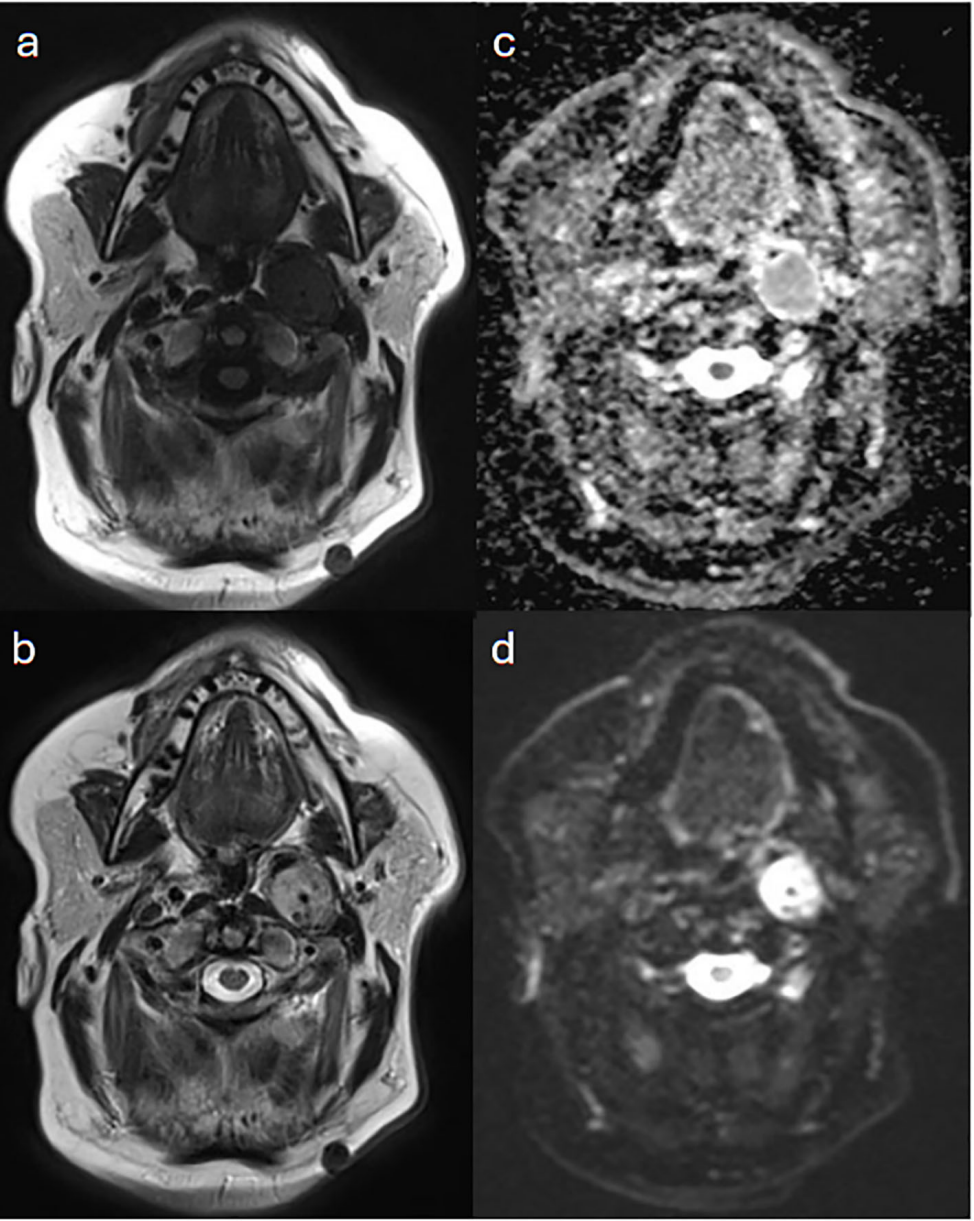
Case 1. A 2.7 cm anteroposterior enhancing carotid space lesion that contains internal flow voids on **(a)** T1-weighted and **(b)** T2-weighted MRI images. The lesion is mildly restrictive to diffusion as seen in **(c)** apparent diffusion coefficient (ADC map) and **(d)** diffusion weighted imaging, to a less extend in comparison with adjacent nodal or neuroaxis soft tissue. The lesion is superior to the carotid bifurcation, displacing both the internal and external carotid arteries anteriorly, and its superior margin is just clear of the jugular foramen. This is consistent with left vagal paraganglioma.

A 70-year-old man presented with abdominal pain and constipation lasting over 6 months. An external CT demonstrated a small bowel lesion, which was looked at in the sarcoma MDT at our institute, raising suspicion of gastrointestinal stromal tumor (GIST); however, carcinoid and neuroendocrine tumors were also in the differential diagnosis. A Ga-68 DOTATATE PET/MRI was requested for further assessment and did not show any increased radiotracer uptake in the mass of concern, but uptake was noted in bilateral neck lesions (**Figure 1**). Head and neck MDT proceeded with a gadolinium-enhanced MRI of the head and neck, revealing a corresponding 4.2 cm craniocaudal enhancing carotid space lesion described with internal flow voids and mild diffusion restriction, consistent with a left vagal PG (**Figure 2**). The abdominal lesion remains under investigation to date.

### Case 2

**Figure 3:**
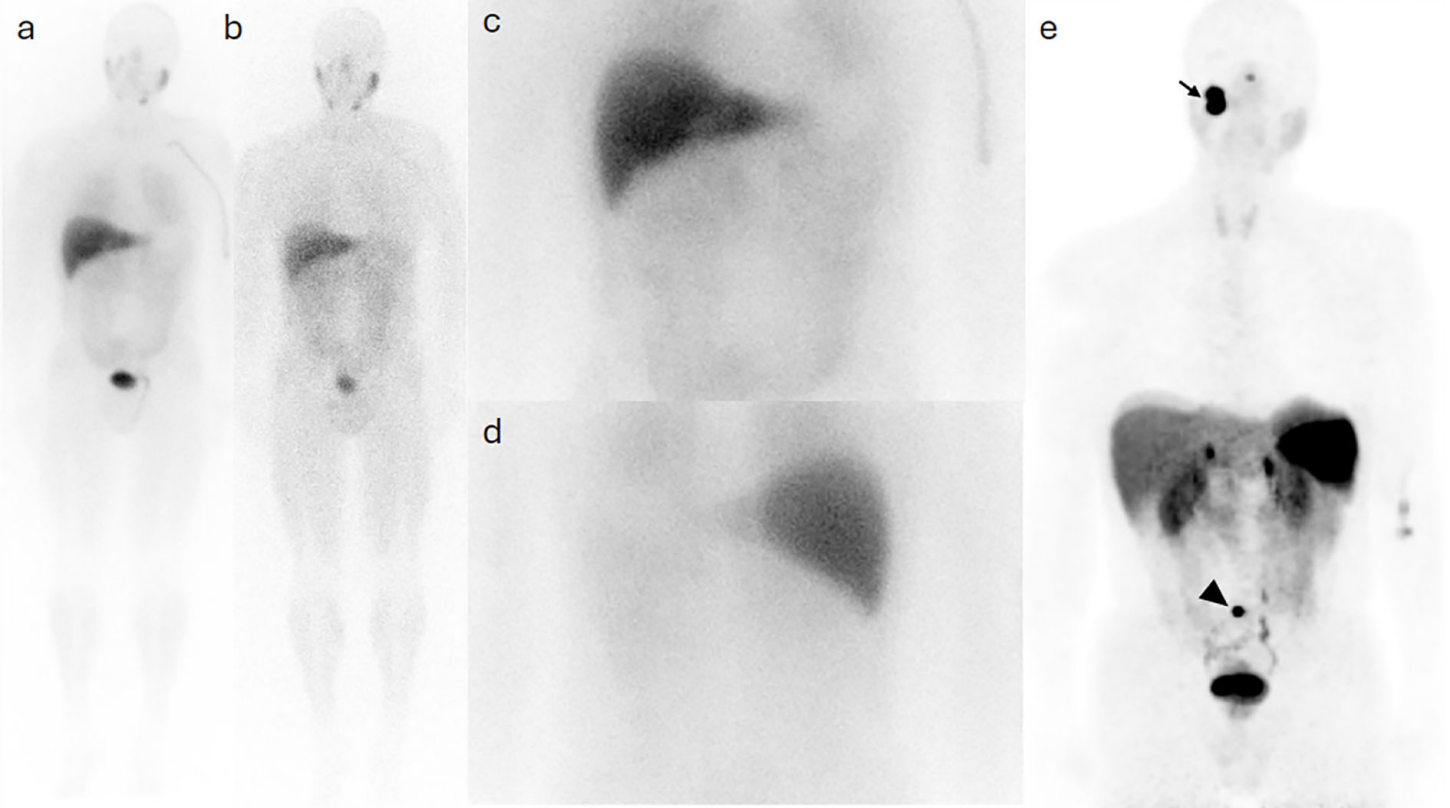
Case 2. I123-MIBG whole body planar images taken at 4 **(a)** and 24 **(b)** hours post injection show minimal activity at the site of the primary disease in the right jugular region, with no evidence of pelvic osseous activity on spot view images of the abdomen and pelvic in an anterior **(c)** and a posterior **(d)** views. The Ga68-DOTA PET MIP image **(e)** demonstrates intense radioactivity at the site of primary lesion (arrow) and a sacral uptake (arrowhead).

A 59-year-old man was known to have left jugular PG, which was resected in 1993 and 2003. He presented with lower back pain, which was thought to be of metastatic nature, given his elevated plasma 3-metho-xytyramine and normal plasma metapherines. Images acquired at 4 and 24 hours post 390 MBq (10.5 mCi) I-123 iodine meta-iodobenzylguanidine (MIBG) showed minimal residual activity at the primary site, with no evidence of metastasis. Subsequently, a Ga-68 DOTATATE PET/CT scan was requested for further assessment. This used 250 MBq (6.75 mCi) with acquisition at 60 minutes post injection and demonstrated intense radiotracer activity correlating to the primary disease, as well as a metastatic focus in the sacrum (**Figure 3**). The sacral metastasis was treated with radiotherapy of 50.4 Gy in 28 fractions, and treatment was successfully completed.

### Case 3

**Figure 4:**
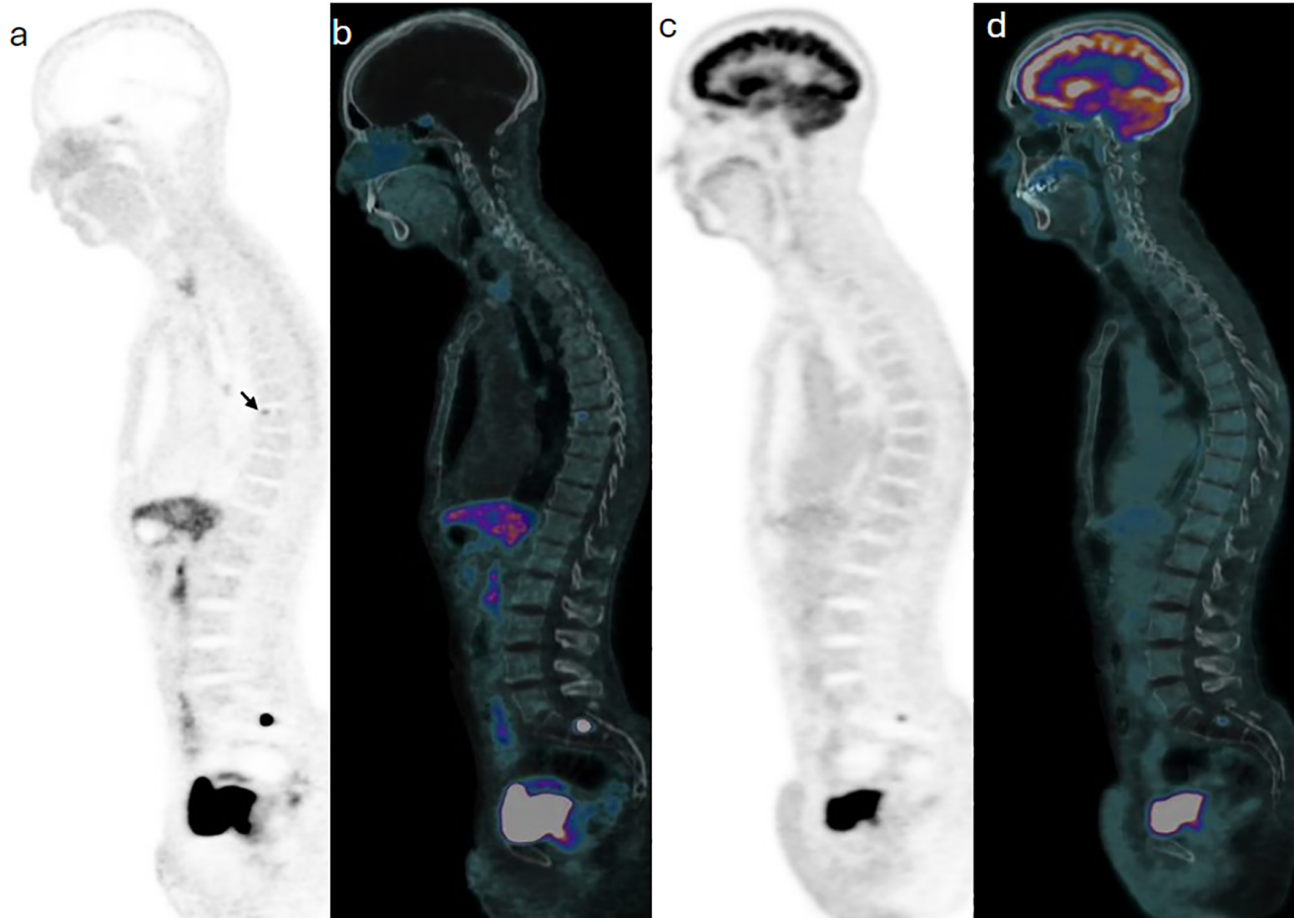
Case 3. Trans-axial view images at the pelvic inlet level show a focal of Ga68-DOTATATE on PET **(a)** and PET/CT **(d)** components of the study, with no correspondent F18-FDG activity on PET **(c)** and PET/CT **(d)** images of subsequent images.

**Figure 5:**
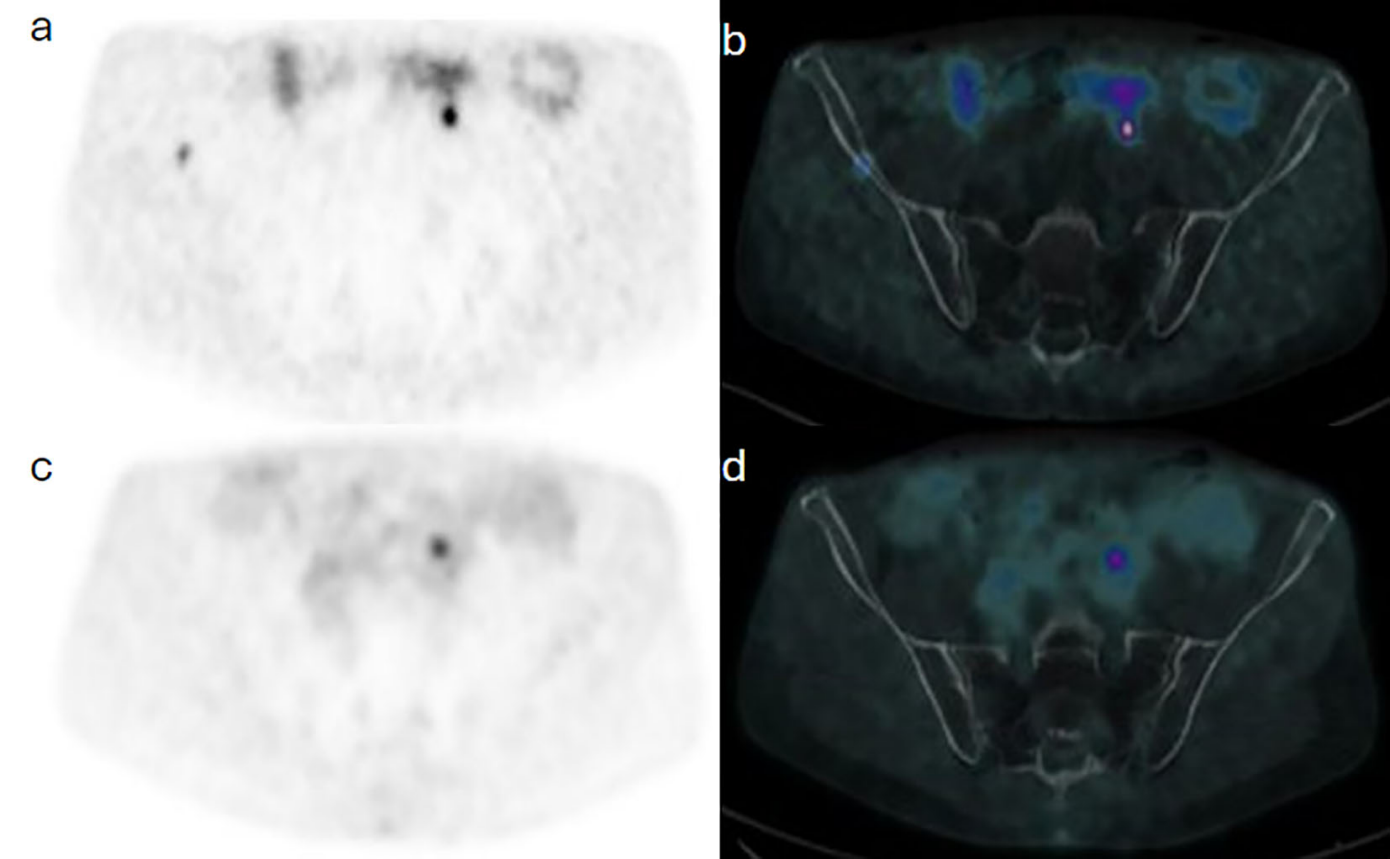
Case 3. Trans-axial view images at the pelvic inlet level show a focal of Ga68-DOTATATE on PET **(a)** and PET/CT **(b)** components of the study, with no correspondent F18-FDG activity on PET **(c)** and PET/CT **(d)** images of subsequent images.

A 64-year-old man with known metastatic jugular PG associated with succinate dehydrogenase (SDH) subtype C gene mutation, which was treated with primary resection and external beam radiotherapy to metastatic bone sites, was referred for annual surveillance. Initially, 435 MBq (11.75 mCi) F-18 fluorodeoxyglucose (FDG) was administered intravenously, and PET/CT was acquired at 57 minutes post injection. Images demonstrated hypermetabolic focal activity at the known metastatic S1 vertebra. Routine blood tests showed a further rise in plasma 3-methoxytyramine from 360 to 446 pmol/L (normal range 0–180 pmol/L). A Ga-68 DOTATATE PET/CT was thereafter done using 173 MBq (4.7 mCi) and acquired at 67 minutes post injection, subsequently demonstrating a corresponding uptake at the known metastatic thoracic and sacral bones (**Figure 4**), as well as new sites of uptake in the right ilium (**Figure 5**).

### Case 4

**Figure 6:**
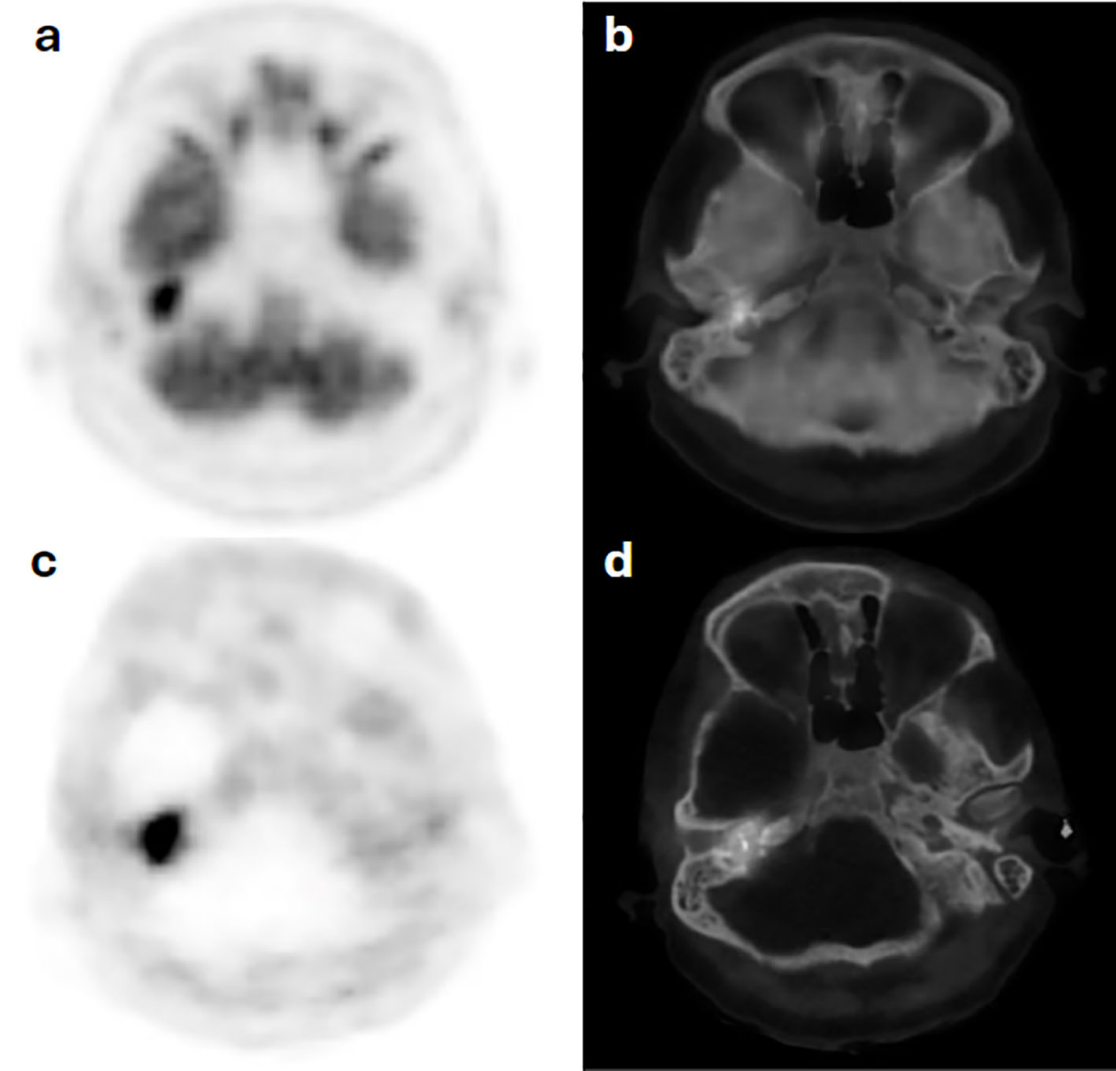
Case 4. F18-FDG PET **(a)** and hybrid PET/CT **(b)** images show increased radiotracer avidity in a tympanic bone destructive lesion on the right side with diffuse subtle avidity along the left auditory canal, which corresponds to similar uptake distribution on Ga68-DOTATATE PET **(c)** and PET/CT **(d)** scan images, not in favor of a glomus tumor on the left tympanic region.

A 67-year-old man diagnosed with bilateral tympanic PG was referred with chronic suppurative otitis media. Clinically, the left external auditory canal was obliterated, with no visualization of the left eardrum. An F-18 FDG PET/CT done using 228 MBq (6.2 mCi) with acquisition performed at 67 minutes post injection revealed a focal area of hypermetabolic activity on the right side and subtle diffuse uptake on the left. To confirm the diagnosis, MDT requested an SSRT assessment. Ga-68 DOTATATE PET/CT was performed using 118 MBq (3.2 mCi) IV, and images were acquired at 63 minutes post injection. The scan showed focal uptake on the right side; however, there was low-grade tracer avidity on the left with diffuse distribution. Consistent functional findings on the left side were not in favor of HNPG, and a revised MRI scan was done, eventually excluding disease (**Figure 6**).

Comprehensive biodemographic and imaging data for all cases are detailed in **Table 1**.

**Table 1:** Summary of demographics, imaging impact and therapy.

Category	Details
Gender	100% Male
Age	Median: 66 years (Range: 59–70 years)
Symptomatic Status	Symptomatic: 50%, with catecholamine secretion
	Non-symptomatic: 50%, non-secreting
Location of Tumours	– Patient 1: Vagal left
	– Patient 2: Jugular left
	– Patient 3: Jugular left
	– Patient 4: Tympanic bilateral
Therapy Adjustment After DOTATATE Finding	100%
Management Modification	– Patient 1: Transition to observational strategy
	– Patient 2: Implementation of radiotherapy
	– Patient 3: Adoption of conservative management due to ineligibility for EBRT^*^
	– Patient 4: Differential treatment approach for right lesion; infection-focused management for left

## Discussion

Our study highlights the role of Ga-68 DOTATATE PET imaging in the management of patients with HNPG.

Despite numerous studies showing the superiority of Ga-68 DOTATATE imaging in detecting small (<1 cm) lesions, with a sensitivity reaching 100% across a wide range of tumor sizes (1, 2, 5, 6) [detecting 36% more lesions than CT and MRI (8, 9)], this modality is still not incorporated into the initial evaluation of HNPGs. In case 1 of our cohort, Ga-68 DOTATATE PET/MRI performed to assess an abdominal mass incidentally detected two lesions suggestive of HNPG. However, a follow-up gadolinium-enhanced MRI was unable to clearly identify the smaller right carotid body PG as a distinct entity. The specific “salt and pepper” feature of HNPG on MRI is known to be less prominent in submetacentric deposition, becoming a source of false negative findings (5, 10). This case illustrates how Ga-68 DOTATATE can influence management decisions; in cases of bilateral HNPG, clinicians may opt for a wait-and-watch approach rather than an immediate surgical excision (5, 11, 12).

Another functional imaging modality is I-123 MIBG, which has been used for imaging phaeochromocytomas and parangliomas. It has also been used in HNPG (3, 6) but with limited success. Despite Ga-68 DOTATATE’s lower detection rate for metastatic lesions compared to primary tumors (13), it still outperforms I-123 MIBG. Studies have shown that I-123 MIBG has limited sensitivity for detecting HNPG metastases, ranging between 19% and 50% (6, 7, 14). In our series, case 2 depicts the superiority of Ga-68 DOTATATE PET imaging in detecting metastatic right carotid body HNPG. In this 59-year-old man, previously treated with surgical resection of a primary tumor twice, an initial I-123 MIBG scan indicated a small area of diffuse activity in the surgical bed only, consistent with local recurrence. Given the patient’s symptoms, a Ga-68 DOTATATE scan, known for its higher detection sensitivity, was subsequently performed. This scan not only confirmed the local recurrence but also revealed an additional focus of increased avidity in the pelvic bone, which lacked a CT morphological correlate, indicating metastatic disease. The detection of a solitary bone lesion aided the use of external beam radiotherapy, eventually revealing a response as evidenced by decreased tumor markers. This case further highlights the limitations of I-123 MIBG, suggesting that it may be more appropriately used in the pre-treatment work-up rather than for comprehensive metastatic evaluation.

Established guidelines recommend the use of either F-18 FDG or Ga-68 DOTATATE for evaluating metastasis in PGs, without preferring one over the other (15, 16). However, this preference is challenged by case 3 in our cohort, with metastatic resected right carotid body HNPG of SDH subtype C mutation. In this case, an annual F-18 FDG PET/CT scan showed a single focus of increased activity at the known pelvic metastatic site, whereas a subsequent Ga-68 DOTATATE PET/CT, performed within 3 weeks of the FDG PET/CT scan because of elevated blood tumor markers, revealed extensive osseous involvement. This suggests that Ga-68 DOTATATE may offer superior sensitivity compared to F-18 FDG for evaluating metastatic disease in this patient group with SDH subtype C mutation. F-18 FDG could potentially be reserved for cases with dedifferentiated disease or lesions associated with SDHx genomic mutations that exhibit higher mitotic rates and increased F-18 FDG affinity (2, 6, 7, 13, 14, 17).

The negative predictive value of Ga-68 DOTATATE for detecting HNPGs is not well established. In case 4 of our cohort—a patient with bilateral HNPG presenting with chronic suppurative otitis media—an F-18 FDG PET/CT revealed focal hypermetabolic activity on the right and subtle diffuse uptake on the left, sites of known disease. Due to the limited specificity of F-18 FDG, a Ga-68 DOTATATE PET/CT was conducted, showing a focal uptake on the right consistent with jugulotympanic HNPG but demonstrating inconclusive results on the left. This case highlights the utility of Ga-68 DOTATATE in potentially ruling out HNPG in the absence of tissue biopsy—which remains the gold standard modality for a definitive diagnosis. In situations where HNPG is suspected based on imaging, Ga-68 DOTATATE PET/CT can assist in excluding the diagnosis if no significant uptake is detected.

## Conclusion

Our case series shows that Ga-68 DOTATATE PET/CT has good potential in the evaluation of HNPGs, offering superior sensitivity for detecting both primary and metastatic lesions compared to other imaging methods. In the context of hereditary disease related to genetic mutations, Ga-68 DOTATATE also shows high performance in disease detection. Its high negative predictive value is particularly useful for ruling out disease. Although Ga-68 DOTATATE is not currently standard for initial diagnosis, its effectiveness in detecting metastases and guiding treatment decisions suggests it should be considered for inclusion in future clinical guidelines. Continued research and the development of standardized interpretation criteria will be essential for its broader adoption and improved diagnostic accuracy.

## Data Availability

The original contributions presented in the study are included in the article/Supplementary Material. Further inquiries can be directed to the corresponding authors.
